# Unusual Mammalian Sex Determination Systems: A Cabinet of Curiosities

**DOI:** 10.3390/genes12111770

**Published:** 2021-11-08

**Authors:** Paul A. Saunders, Frédéric Veyrunes

**Affiliations:** 1 Institut des Sciences de l’Evolution de Montpellier, ISEM UMR 5554 (CNRS/Université Montpellier/IRD/EPHE), 34090 Montpellier, France; frederic.veyrunes@umontpellier.fr; 2 School of Natural Sciences, University of Tasmania, Sandy Bay, TAS 7000, Australia

**Keywords:** mammals, rodents, sex determination, sex chromosomes, meiosis

## Abstract

Therian mammals have among the oldest and most conserved sex-determining systems known to date. Any deviation from the standard XX/XY mammalian sex chromosome constitution usually leads to sterility or poor fertility, due to the high differentiation and specialization of the X and Y chromosomes. Nevertheless, a handful of rodents harbor so-called unusual sex-determining systems. While in some species, fertile XY females are found, some others have completely lost their Y chromosome. These atypical species have fascinated researchers for over 60 years, and constitute unique natural models for the study of fundamental processes involved in sex determination in mammals and vertebrates. In this article, we review current knowledge of these species, discuss their similarities and differences, and attempt to expose how the study of their exceptional sex-determining systems can further our understanding of general processes involved in sex chromosome and sex determination evolution.

## 1. Introduction

Sex determination is a fundamental process in all eukaryotes with separate sexes [1]. In many species, sex is determined genetically, by the expression of one or several genes carried by sex chromosomes. The most common genetic sex-determining systems among vertebrates are male heterogamety, whereby females are XX and males XY, and female heterogamety, with ZW females and ZZ males. Sex chromosomes have evolved independently many times, and are understood to emerge once a member of an autosomal pair acquires a sex-determining gene or allele. How often do new sex chromosomes evolve? As it turns out, there is a striking dichotomy between taxa that undergo frequent sex chromosome turnover (i.e., change in location and/or identity of the sex-determining gene), as observed in many fish and amphibians [2,3], and groups that have old and conserved sex chromosomes. The X and Y of therian mammals (placentals and marsupials) are a textbook example of this second category. They appeared around 150 My ago [4], when *Sry*, the mammalian male sex-determining gene [5], arose on an autosome. Following loss of recombination along most of their length, they diverged gradually over time [6]. Nowadays, the X and Y of most mammals share only a tiny homologous region: the pseudo-autosomal region (PAR), and the rest of the pair is completely dissimilar in sequence and structure. The X chromosome is large and gene-rich, and the Y is small and has lost most of its ancestral gene content: the only genes that have survived the degeneration process are *Sry* and a few other genes that play essential roles in spermatogenesis and male fertility, and dose sensitive genes [7,8,9].

Old sex-determining systems with highly heteromorphic sex chromosomes are considered to be “evolutionary traps” [10], meaning that it seems that once a certain stage of sex chromosome differentiation and specialization has been reached, sex determination is locked-in, i.e., changing the mechanism of sex determination becomes near impossible. This is due to the production of lethal genotypes (such as YY), to the requirement of specialized Y-linked genes in the production of males, and conversely, the potential adverse effects of these same genes in the production of females, and finally, to the reduction in fertility due to meiotic impairments. Indeed, any deviation from the standard XX female/XY male system generally leads to sterility or very poor fertility, as illustrated by Klinefelter (XXY) and Turner (XO) syndromes in humans [11,12], and sex-reversed individuals (XX males or XY females) in mice and domestic animals [13].

However, rules are meant to be broken, and mammalian sex determination is no exception. Around a dozen therian mammals have been described with so-called “bizarre” and “weird” sex-determining systems [14,15] which deviate from the standard therian mammal XX/XY mechanism. These species, all rodents, have puzzled scientists for nearly 60 years, since Robert Matthey, who characterized a fair share of these atypical systems, analyzed karyotypes of the mole vole *Ellobius lutescens,* in which the Y chromosome is missing [16]. Since then, a few more species with no apparent Y have been identified, and in a few others, fertile XY females have been observed alongside XY males and XX females. All these species defy what is considered to be the norm in mammals, and can be viewed as weird phenomena of nature. However, as Jenny Graves argued “some of the most informative new observations on vertebrate sex and sex chromosomes have come from animals that are far from being models” [17], and recently, the detailed analysis of meiotic sex chromosome behavior in some rodents with unusual sex-determining systems has furthered our understanding of the relationship between sex chromosome evolution and meiosis in mammals [18,19]. 

Overall, mammalian species with bizarre sex-determining systems have been poorly studied. Nevertheless, a sizeable body of new literature has appeared since the last comprehensive review on these species, almost 30 years ago [14]. New weird sex-determining systems have been discovered, others have been revised, and our understanding of the ultimate and proximate mechanisms involved in their evolution has grown. In this paper, we attempt to present an updated critical review of the literature published about these species. We deliberately excluded the multiple sex chromosome system of monotremes, which has an independent origin from the rest of mammals, and was reviewed recently [20,21], as well as other mammalian species with sex-autosome fusions (e.g., XX/XY_1_Y_2_ or X_1_X_1_X_2_X_2_/X_1_X_2_Y systems), as the latter does not involve an alteration of the mechanism of sex determination per se. We first make a detailed overview of work published on the species that have received the most attention. We divided these species in two categories, those in which XY females are found alongside XX females and XY males, and those in which the Y chromosome is missing. Then, we summarize information known about other species which have received less attention and/or in which the unconventional nature of sex determination is still uncertain. Finally, we discuss the similarities and differences found among these odd systems, the hypotheses made to explain how they evolved, and address how unravelling their complexity could help further our understanding of general aspects of mammalian sex chromosome stability, organization, function, and evolution.

## 2. Species with Y-Bearing Females

Sporadic XY sex reversal cases have been reported in a large variety of mammalian species, including humans. They are generally associated with reduced fertility or sterility, along with other symptoms (for a review in humans, see [22]; and for other mammals, see [13]). XY male-to-female sex reversal has been extensively studied in laboratory strain mice, where the major causes of reduced fertility are the presence of a single asynapsed X chromosome and the ectopic expression of Y-linked genes during meiosis, leading to increased oocyte loss (e.g., [23,24,25,26]). In females that manage to bypass these meiotic constraints, fertility is still expected to be reduced as 25% of embryos produced (YY) are unviable. Nevertheless, sex determination systems with a high proportion of fully fertile XY females have evolved at least five time independently in mammals. 

### 2.1. Myopus Schisticolor

The wood lemming *M. schisticolor* (Arvicolinae, Rodentia) is the first mammalian species ever described with fertile XY females. The Y chromosome of these females is identical to that of males, and it is actually the X that causes sex-reversal: they carry a special variant of the X (called X*) which suppresses the male-determining function of the Y (*Sry* is supposedly still present and active on the Y [27,28,29]). Thus, there are three kinds of females, XX, XX*, and X*Y, while males are all XY [30,31]. Chromosome bandings, FISH techniques, and meiosis analyses have revealed the presence of a deletion of about 1 Mbp in the X_p21–23_ region, as well as an inversion in the short arm, that changed the wild-type X chromosome into the derived X* [28,32]. Molecular analyses identified the *Cct7* gene, closely located to the deleted region and mainly expressed in testis, as a potential candidate for the sex-reversal mutation [27]. However, more recent studies refuted this hypothesis: this gene is autosomal and has undergone intense duplication activity in vertebrates, with pseudogene sequences found on the X chromosome (e.g., [33]). To this day, it is still unknown which gene causes feminization of X*Y individuals. As one could expect, the sex chromosome system of the wood lemming leads to a distorted sex ratio in favor of females. For instance, in a Siberian population, a large excess of females was observed, associated with a high frequency of X*Y females [34]. Whereas XX and XX* females produce sons and daughters in a 1:1 ratio (XY sons and XX daughters) and 1:3 ratio (XY sons, and XX, XX* and X*Y daughters), respectively, in line with Mendelian segregation expectations, X*Y females give birth to females only (XX* and X*Y, in equal number). This is achieved by a mechanism of double non-disjunction of early oogonia in fetal ovaries, that leads to the formation of YY and X*X* primary oocytes, where the former, which do not contain essential X-linked genes, degenerate. Thus, only X*-carrying eggs are produced, zygotes develop into XX* or X*Y females, and as such, no costly YY embryos are produced [28,30,35]. Consequently, the fertility of the X*Y females is not reduced compared to that of other females. Surprisingly, it was shown that these females actually have a higher fertility, owing to a greater proportion of growing oocytes, an earlier onset of reproduction, and higher pregnancy rate [29,36]. Notwithstanding, female meiosis analyses have shown that X*Y females have a much higher frequency of abnormal oocytes than other females, with univalent configuration particularly common, probably due to the complex double nondisjunction mechanism [28]. Occasional failure of this mechanism in X*Y females leads to the relatively frequent occurrence (around 2%) of specimens with numerical sex chromosome aberrations, including X0 females, X*YY females and XX*Y individuals, while no autosomal trisomy or monosomy has ever been found [37,38,39]. Neither the X0 constitution nor the presence of an extra Y chromosome in females preclude fertility, however XXY males are always sterile [38]. Interestingly, among the specimens with XX*Y sex genotype, the authors found one male, one female, and one hermaphrodite, which displayed different patterns of X inactivation. In the male, the X* was inactivated in all cells inspected, while in the female, it was the X that was systematically inactivated. In the hermaphrodite, an X/X* inactivation mosaicism was suspected [39].

### 2.2. Dicrostonyx Torquatus

The collared lemming *D. torquatus* (Arvicolinae, Rodentia) has a sex determination system similar to the one of the wood lemming, with females being XX or XY owing to an X-linked mutation [14,40,41,42], although these two species are not closely related [43]. However, in contrast to *M. schisticolor*, the X and mutant X* chromosomes cannot be distinguished by conventional cytogenetic methods, and the Y chromosome is not easy to identify due to a series of translocations between autosomes and sex chromosomes [42]. This has resulted in misinterpretations: in several papers, the system is described as being made up of XX and X0 females and X0 males (e.g., [44,45]). In addition, the X*Y females have not evolved the double non-disjunction mechanism observed in *M. schisticolor*: they produce both X*- and Y-carrying eggs. Hence, theoretically, the litter size of X*Y females should be reduced by one-quarter due to the loss of YY embryos, but owing to a higher ovulation rate, their reproductive success is not significantly lower than that of females with two X chromosomes [14,46]. Finally, the population sex ratio is lower and proportion of X*Y females higher than expected in both natural and captive colonies [34,47]. Gileva demonstrated that this deviation could be explained by a male sex chromosome drive (non-Mendelian transmission of sex chromosomes), as collared lemming males in her laboratory colony transmitted their Y chromosome at a rate of 54–59 [48].

### 2.3. Akodon Species

The sex determination of the South American field mice *Akodon* (Sigmodontinae, Rodentia) is a complex story. The existence of fertile females with heterogametic sex chromosomes indistinguishable from that of males (living alongside standard homogametic females) was first reported more than fifty years ago in *A. azarae* [49]. The “Y-like” chromosome was initially described as a largely deleted and heterochromatic X chromosome (called *dX* or *x* in different papers; [50,51]), but in the eighties and early nineties, different studies, and notably the discovery of *Sry* in variant females, revealed unambiguously that these females are in fact XY [52,53,54]. For twenty more years, cytogenetic, molecular, breeding, and phylogenetic studies assumed the sex-reversal mutation was on the Y chromosome (e.g., [50,53,54,55,56]), a hypothesis that led to questionable conclusions (e.g., [57,58]), as in 2009, the analysis of C-banding patterns and hereditary transmission discarded the possibility that the sex-reversal mutation is carried by the Y, supporting instead a causal role of an X-borne mutation, as in *M. schisticolor* and *D. torquatus* [59]. At least nine species of *Akodon* have been reported with XY females, with a prevalence of said females varying from 10 to 60% [56]. The mitochondrial-based phylogenetic reconstructions were inconclusive to distinguish between a single or recurrent origin of sex-reversal [57]. As in *D. torquatus*, *Akodon* XY females produce X and Y oocytes [51], therefore, their litter size should be significantly lower than that of XX females. However, normal and XY *A. azarae* females exhibit similar litter sizes, indicating that the variant females have acquired a compensation mechanism for the loss of YY embryos [56]. In this regard, Lizarralde et al. detected a higher ovulation rate in XY females [50], although this finding could not be replicated by Espinosa and Vitullo, who nevertheless found that XY females have a longer reproductive lifespan than XX females [55]. Sex chromosome meiotic behavior of *A. azarae* XY females has been investigated and shows a noticeable difference from that of XY males [51]. The X and Y either pair in a small terminal region, the remaining asynapsed regions becoming transcriptionally silenced as in males (in 40% of the cells), or they are engaged in extensive self-synapsis (in 60% of the cells). This degree of self-synapsis is such that the two chromosomes can be totally unpaired and both fully self-synapsed. Finally, data on sex ratio of litters from two *A. azarae* laboratory colonies suggest the presence of Y-drive in XY females (63%) and potentially in males too (55%) [58], though these findings might need to be revised, as they were published assuming sex-reversal is due to a mutant Y chromosome, rather than a feminizing X.

### 2.4. Mus Minutoides

The African pygmy mouse, *Mus minutoides* (Rodentia; Murinae) is the latest addition to the short list of mammals with odd sex determining systems. As in the species discussed above, it is characterized by a very high proportion of sex-reversed females (up to 60% of the females in some populations) due to a feminizing X* chromosome, which emerged at least 0.9 million years ago [60,61]. The two Xs of this species are easily told apart cytogenetically, owing to successive rearrangements, and are randomly inactivated in XX* females [62]. *M. minutoides* belongs to the subgenus *Nannomys*, known for its exceptional karyotypic variability [63]. Among other remarkable features, *Nannomys* mice are characterized by the greatest diversity of sex-autosome translocations identified in mammals to date [64]. In *M. minutoides,* sex chromosomes are fused to the largest autosome of the genome [65,66]. However, in some populations, the sex chromosomes have undergone further rearrangements: in eastern Africa, the X is unfused, probably due to a subsequent fission [67], and in one South African population, the X* is fused to another autosome due to a whole arm reciprocal translocation event (WART) [64]. Another rare feature in *Nannomys* is the absence of PAR: the X and Y do not pair nor form chiasmata during male meiosis [68,69,70]. In the African pygmy mouse, the sex-autosome translocations have nevertheless restored a large segment of homology between the X and the Y: a neo-PAR, where synapsis and DNA repair dynamics are disturbed and recombination largely suppressed, revealing early signs of sexual differentiation of this region [18]. Female meiosis has been investigated in the South African population that has undergone a WART [25]. This chromosomal rearrangement has led to complex meiotic configurations in XX* and X*Y females, with sex chromosomes involved in quadrivalents. Meiosis is further impeded in the latter genotype by the presence of a single X and the Y chromosome, and by insufficient numbers of crossovers along sex and neo-sex chromosomes and frequent non-homologous synapsis. Despite all this, X*Y females are overrepresented in this population and fully fertile. Histological studies have shown that these females display typical ovaries, with no sign of testicular tissue [71], and the comparison of life history traits in a laboratory colony revealed that, against all odds, they have an enhanced fertility compared to XX and XX* females, due in particular to an earlier onset of reproduction and larger litter size, despite the loss of YY embryos [72]. Nevertheless, X*Y females display some masculinized features, such as enhanced aggressiveness, lower anxiogenic response [73] and higher bite force performance [74], suggesting that the effect of the presence of the X* goes well beyond sex reversal only, and is at the origin of a third, complex, sex phenotype. The expression of some of the main genes involved in male and female differentiation was investigated, revealing in particular that (i) *Sry* is expressed both in X*Y female brain and gonads, and (ii) that the X-linked gene *Nr0b1* (*Dax1*), known for inducing male-to-female sex reversal when duplicated in humans, shows no sign of changes in either copy number or expression on the X* [71]. The gene responsible for sex-reversal is still unknown, but based on in vitro experiments, Zhao et al. suggested that the degraded *M. minutoides Sry* sequence has weakened the activity of the gene and compromised the entire SRY/SF1/TESCO nexus [75], which could have favored the rise of an X*-based feminizing mechanism. However, this result needs to be reevaluated in the light of recent findings on the presence of a second exon in mouse *Sry* that is crucial for male development [76], and on the questionable critical role of TESCO (the testis-specific enhancer to which *Sry* binds to upregulate *Sox9*) [77]. Finally, it was recently revealed that the three types of females produce more males than expected assuming a Mendelian inheritance of sex chromosomes. This sex ratio bias is due to a strong transmission distortion of male sex chromosomes, which surprisingly varies along with female genotype: males transmit their Y more often (80% transmission rate) in crosses with XX and XX* females, and less often (36%) in crosses with X*Y females. Mathematical models suggest that this conditional drive of the male sex chromosomes protects the X* against loss [78]. 

### 2.5. Lasiopodomys Mandarinus

There is still a lot to discover in this species. The mandarin vole *L. mandarinus* (Arvicolinae, Rodentia) is arguably one of the most enduring puzzles of mammalian sex chromosome systems. Initially, females from a Russian population were reported as being either XX or X0, and males XY [79]. This observation was corroborated by the study of several Chinese populations [80]. Two morphologically different Xs (that vary in length, centromere position, and G-banding patterns) were observed in these populations, one always associated with X0 females, and another with males [80,81,82]. Intriguingly, the presence of the ancestral Y chromosome and *Sry* was declared uncertain, in particular following the failure to amplify the *Sry* HMG-box (highly conserved across mammals), with primers designed to amplify human/mouse/vole *Sry* [83]. At first, the hypothesis of a Y-loss in *L. mandarinus* seems in total contradiction with the description of males with heterogametic sex chromosomes. However, chromosome painting analyses revealed that the sex chromosomes have fused with autosomes [84], and segregation studies show that one chromosome segregates as expected from a Y [85]. Whether this chromosome consists of the association of the ancestral Y (whole or fragmented) and a neo-Y, or is just composed of a neo-Y (following complete loss of the ancestral Y) remains to be determined. For simplicity, we will refer to this chromosome as the “Y” thereafter. Based on cross-breeding experiments, Romanenko et al. studied the transmission patterns of sex and neo-sex chromosomes and revised the sex-determining system of the mandarin vole: they describe one type of males (heterogametic), and three distinct female “karyomorphs”: a homogametic one, a heterogametic one with the two morphologically different Xs, and a heterogametic one with the female specific X and the Y [85]. As mentioned by the authors, the pattern of association between phenotypic sex and sex chromosome combinations is highly reminiscent of the ones found in other rodents with X*Y females, and the change in sex determination of the mandarin vole fits well with the existence of an X-linked feminizing mutation. However, the failure to detect any Y-specific genes or regions may suggest that this system has gone an evolutionary step further, with the loss of the ancestral Y after it became bisexually transmitted. Finally, Romanenko et al. also noticed that the proportion of each genotype sired by females was inconsistent with a standard Mendelian inheritance of sex chromosome [85]. Roy reanalyzed this data and suggested that it could be explained by the presence of a male Y-drive in crosses with XX females, and male X-drive in crosses with XY females [86], which would make this drive system identical to the conditional drive described in the African pygmy mouse. 

### 2.6. Conclusions

Naturally occurring XY sex reversal with no apparent impact on female fertility has evolved at least five times independently in mammals: twice in lemmings, in the wood and collared lemmings *M. schisticolor* and *D. torquatus*; in several species of South American field mice of the genus *Akodon*, where it is still not clear if the sex reversal condition appeared once or multiple times [57]; in the African pygmy mouse *M. minutoides*; and in the Mandarin vole *L. mandarinus*. Surprisingly, the five models share several common features, and the most intriguing is that in all cases, sex reversal is not due to a mutation of the *Sry* gene or any other Y-linked genes, but to the presence of a third sex chromosome, a mutant of the X, called X*, that supposedly blocks the male program initiated by the Y chromosome. This happens even in *Akodon* species, in which sex reversal was previously misinterpreted as being caused by a loss of function of the Y chromosome (see [59]). These results strongly suggest that the mammalian X chromosome carries one or several still unknown genes necessary for the mammalian sex determination program, and which may lead to the evolution of feminizing X* chromosomes when mutated. Other common features are discussed below. 

## 3. Species That Have Lost the Y

Mammalian sex chromosomes, Y in particular, have been a source of fascination for many years [6]. As they evolved from a pair of autosomes, they have undergone dramatic evolutionary changes. While the X conserved most of its ancestral gene content, the Y chromosome has undergone a drastic process of degeneration and has retained only a handful of genes. Why the Y persists in mammals is still debated to this day (e.g., [87]), but all models proposed to date agree on one thing: the Y carries several genes, shared across all (or most) mammals [7,88,89], that play a primordial role in male function and fertility: the sex-determining gene *Sry* of course, and other genes as such as *Zfy*, *Eif2s3y*, and *Rbmy* [9,90,91]. Nevertheless, a few mammalian species have lost their Y, suggesting that the male-specific sex chromosome may not be invulnerable.

### 3.1. Ellobius Species

The transcaucasian mole vole *Ellobius lutescens* (Cricetidae, Rodentia) was the very first mammal in which a sex chromosome complement deviating from the standard XX/XY was described. Both males and females carry an odd number of chromosomes (2*n* = 17), and with no apparent Y chromosome, Robert Matthey concluded this species exhibits an X0/X0 sex-determining system [16,92]. As it turns out, other mole voles were later found to have lost their Y chromosome too: while the sister species of *E. lutescens*, *E. fuscocapillus* has a normal XX/XY sex-determining system, the three other species of the genus (which belong to another subgenera), *E. talpinus*, *E. tancrei*, and *E. alaicus*, all have an XX/XX sex-determining system, unique in mammals (see [93] for a detailed review of the discovery and description of these systems). This suggests two independent losses of the Y chromosome in the genus. A few years after the discovery of the mammalian sex-determining gene *Sry* [5], Just and his collaborators revealed that the gene is completely absent in *E. lutescens* and *E. tancrei* [94]. It was later shown it is also missing in the two other species with no Y chromosome [95,96], and while fragments of *Sry* were detected in *E. fuscocapillus*, it is unclear whether it still plays its testis determining role in this species, as it is present in multiple degenerated copies in both sexes [96]. The loss of function of *Sry* has been speculated to be linked to a deletion in its target TESCO in the common ancestor of mole voles [97]. To this day, it is still unknown which gene has taken over the role of the switch at the top of the sex-determining cascade, and none of the following genes (involved in testis and/or ovarian differentiation) show an association with sex: *Sox9*, *sf1*, *Sox3*, *Atrx*, *Nr0b1*, *Ar*, *Foxl2*, and *Dmrt1* [94,98,99,100,101]. Despite the loss of the Y and *Sry* in XX/XX and X0/X0 mole voles, several usually Y-linked genes (*Ssty* in *E. tancrei* and *E. talpinus*, *Usp9y* in *E. lutescens*, and *Zfy* and *Eif2s3y* in both lineages) were identified in both male and female genomes: they were saved due to independent relocations to other parts of the genome, presumably the X chromosome [95,96]. Incidentally, the X chromosome(s) carried by males and females are totally isomorphic, i.e., they appear to be completely undifferentiated, and no karyological nor genomic differences have been identified to date [95,99,102]. Whether or not an X chromosome carries the new sex determiner remains to be confirmed. A substantial part of the research devoted to mole vole sex determination has focused on meiosis, as they violate several “rules” that seem to apply in other mammals, i.e., features that are essential to ensure a successful meiotic division (the Y chromosome is necessary during male meiosis, female meiosis requires two active Xs for completion etc.). In *E. lutescens*, the lone X remains unpaired during meiosis in both sexes, and has retained features typical of mammalian XY meiosis (e.g., transcriptional silencing of the X [92,102,103]). Despite successful meiotic reduction, this species faces large embryonic mortality (50%) [104], due to the early loss of zygotes with no sex chromosomes (00) and those with two Xs (possibly due to a lack of X inactivation [99]). In the XX/XX species (*E. talpinus*, *E. tancrei*, and *E. alaicus*), synapsis of XX sex chromosomes is restricted to small telomeric regions in males, in spite of the two Xs being completely homomorphic [96,105,106]. More recently, it was demonstrated that recombination is greatly reduced (possibly absent) in the central region of the XX pair in both sexes of *E. tancrei*, and that one of the two Xs in males displays structural and epigenetic modifications [19], supporting the idea that one of the two Xs carried by males might have acquired a new male-determining role. Finally, in *E. fuscocapillus*, the only mole vole with an XX/XY system, the dynamics of meiosis are also modified: the X and Y first completely synapse at zygotene, before undergoing early desynapsis at pachytene [96]. Taken together, all these findings suggest that meiotic instability might be a trait inherited from the common ancestor of all mole voles, and may have played a role in the evolution of the unusual sex-determining systems found in this group.

### 3.2. Tokudaia Osimensis and T. tokunoshimensis

The *Tokudaia* genus (Murinae, Rodentia) consists of three species, each endemic to a small island in the Ryukyu archipelago between Japan and Taiwan [107,108]. Due to their endangered status (IUCN Red List; https://www.iucnredlist.org/, accessed on 8 October 2021), research on these species has been unfortunately limited. Similar to *E. lutescens*, *T. osimensis* and *T. tokunoshimensis*, found, respectively, on Amami and Tokunoshima islands, have an odd number of chromosomes (2*n* = 25 and 2*n* = 45, respectively), and display an X0/X0 sex determination system, due to the loss of the Y chromosome in their common ancestor [109,110]. No karyotypic differences between sexes have been detected, suggesting that the sex-specific region is extremely small in these species [111,112]. As in *Ellobius* voles, the complete disappearance of the Y chromosome coincides with the loss of the sex-determining gene *Sry* [113,114], but not of other Y-linked genes with important spermatogenesis function (e.g., *Tspy*, *Zfy*, *Eif2s3y*, and *Kdm5d*), which have been translocated or transposed from the Y to the X and/or autosomes, and are therefore present in both male and female genomes [115,116]. Nevertheless, not all ancestrally Y-linked genes were saved, as at least one of them, *Rbmy1a1*, was lost alongside *Sry* [116]. The loss of *Sry* has had consequences on the top of the sex-determining cascade, including the loss of function of TESCO [117] (see also [118,119]). The gene that has taken the place of *Sry* remains unknown. It has been suggested that *Cbx2*, which is found in more copies in males than females and which loss of function causes XY sex reversal in mice and humans, or *Er71*, another enhancer of *Sox9*, could be guiding testes formation instead of *Sry* [118,120], but this remains to be confirmed. Intriguingly, sex determination in the third species of the genus, *T. muenninki*, despite its standard XX/XY karyotype (2*n* = 46), might also be independent of *Sry*, and has been proposed to be in an intermediate state of Y loss. Most of its Y-linked genes have undergone massive amplification and translocation events (including translocations to the X [121]). The original copies of several of these genes (*Tspy*, *Eif2s3y*, and *Usp9y*) are non-functional, but some translocated copies are. Similarly, most copies of *Sry*, found in around 70 copies along the Y chromosome, are non-functional. The most conserved copy, which shows transcriptional activity, was unable to cause testis development in *Sry+* transgenic XX mice [122,123], casting doubt on its role as the initial switch for sex determination in this species. If *Sry* had lost its function, the Y chromosome of *T. muenninki* could meet the same fate as the Y of *T. osimensis* and *T. tokunoshimensis*. Nevertheless, a crucial difference exists between their sex chromosomes: in *T. muenninki*, the X and Y are fused to a pair of autosomes, which, it has been speculated, may have saved the Y from loss [124].

### 3.3. Microtus Oregoni

Sex determination in the creeping vole *Microtus oregoni* (Arvicolinae, Rodentia), another species with no Y, has received less attention than the two others. The unusual nature of its sex chromosome complement was also discovered by Robert Matthey a few years after the one of *E. lutescens*, and was initially described as analogous to the sex-determining system of the latter [125,126]. A few years later, Ohno and his colleagues revised this interpretation, proposing the existence in *M. oregoni* of an XY/X0 system rather than an X0/X0 system. In addition, they revealed that both sexes in this species are gonosomic mosaics, i.e., sex chromosome complements are different in germinal and somatic cell lines [127,128]. Following their terminology, somatic cells are XY in males and X0 in females, while gonadal cells are Y0 and XX, respectively. This discrepancy was explained by a selective non-disjunction of sex chromosomes in the germline of both sexes. This phenomenon results in only two possible combinations at fertilization: XY and X0, and therefore precludes the production of XX females, and non-viable Y0 embryos. A recent study focusing on the genomic constitution and origin of sex chromosomes in *M. oregoni* has once again transformed our understanding of this system. As it turns out, both sex chromosomes (Ohno’s X and Y chromosomes) actually have a chimeric origin: they both derive from the ancestral mammalian X chromosome, and both carry core ancestral Y genes (*Ddx3y*, *Eif2s3y*, *Kdm5d*, *Sry*, *Ube1y*, *Usp9y*, *Uty*, *Zfy*, and *Rbmy*), likely due to an X–Y fusion [129]. The authors of this new study proposed new names for the sex chromosomes, reflecting their major ancestry: X^P^ for the paternally transmitted, male specific sex chromosome and X^M^ for the maternally transmitted chromosome found in both sexes (making it an X^P^X^M^/X^M^0 system). X^P^ and X^M^ are largely collinear, but exhibit subtle differences, especially in the region of Y origin. Females are exposed to Y-derived genes that seem mostly intact and functional, including *Sry*, found in multiple copies on both chromosomes. How sex is determined in this species remains unknown. Among the other bizarre features of this system is the X inactivation (typical of females in other mammals) which occurs in males and is non-random, as inactivation affects the paternally transmitted X^P^ chromosome exclusively, at least in the somatic line [129]. For now, the steps leading to the evolution of this exceptional system from a standard mammalian sex-determining system remain a mystery. Two decades ago, Charlesworth and Dempsey proposed a theoretical explanation for the evolution of the XY/X0 system of *M. oregoni*, involving the spread of mutant X with complete transmission advantage in females and complete disadvantage in males [130]. The recent discovery of the chimeric nature of sex chromosomes in this species suggests a more complex origin. 

### 3.4. Conclusions

In sharp contrast with the idea that therian mammal sex chromosomes constitute an evolutionary trap, the Y chromosome has been lost independently at least four times in various rodent species with fully fertile males: twice in mole vole species of the genus *Ellobius*, in Japanese spiny rats of the genus *Tokudaia*, and in the creeping vole *Microtus oregoni* (see also the next section). These odd systems show common features, suggesting that Y-loss has followed somewhat the same path. First, though the Y chromosome as a physical entity is absent, it has not completely vanished from their genomes. Some Y-limited genes, highly conserved across mammals and deemed essential for male fertility (e.g., *Tspy*, *Zfy*, and *Eif2s3y*), have escaped loss through independent translocations to other regions of the genome. This indicates that retention of these genes is more important than preserving an entire sex chromosome; the Y chromosome could probably not have disappeared without these translocations. Second, in mole voles and spiny rats that have lost their Y, the gene that normally initiates the male developmental pathway in mammals, *Sry*, is absent. In the creeping vole, it is present in both sexes (due to a translocation), casting doubt on its role of primary switch for sex determination. What this implies is that these species (at least the former), have undergone a proper, complete transition to a new sex-determining mechanism. Which gene(s) has/have taken *Sry*’s spot at the top of the sex-determining cascade remains unknown to this day.

## 4. Other Species

Unusual sex chromosome constitutions have been reported in a few other mammalian species. Most of them have been very poorly studied (reported observations are often limited to only a few karotypes), and their status needs to be confirmed as observing a deviant sex chromosome number or constitution does not necessarily equate to a change in the sex-determining mechanism per se. This is well illustrated by members of the marsupial *Peramelidae* family, i.e., the bandicoots. In at least four genera, one X chromosome in females and the Y chromosome in males can be eliminated from somatic cells of different tissues at different stages of development. This results in a sex chromosome mosaicism: with XX and X0 cells in females and XY and X0 cells in males. This has been interpreted as an extreme form of X inactivation and Y elimination in soma while maintaining a standard XX/XY system in the germ line [131,132]. Likewise, most karyotyped females in the two-toed sloths *Choloepus hoffmanni* and *C. didactylus* were described as carrying a single X [133,134,135]. The authors hypothesized that it may just represent a mechanism similar to that observed in the aforementioned marsupials with a complete elimination of the inactivated X in somatic tissues, but this remains to be confirmed, as female meiosis has never been investigated. A spiny mouse species of the genus *Acomys* shows another interesting sex chromosome constitution. Formerly known as *Acomys selousi* [136,137], a taxonomic revision recognized this species as *A. ngurui* [138]. First characterized by Matthey [136], females are X0 with a single giant X chromosome, whereas males are mosaics with X0/XY cells in somatic tissues, and XY cells in the germ line [137,138,139]. Female meiosis has not been characterized, but the occurrence of one female with a small proportion of cells with two X chromosomes has led Castiglia et al. to support the hypothesis of a post-meiotic elimination of one X chromosome [139], as in marsupials and sloths. Based on karyological data, *A. ngurui* was assumed to be living in sympatry with the morphologically very similar *A. spinosissimus*, that presents a classical XX/XY sex chromosome system, the two species only being separated by their karyotypes. However, cytochrome b gene sequencing revealed that both types of specimens share the same haplotype and form a unique species [138,140]. The authors of these studies concluded that *A. ngurui* has a standard sex determination system, XX/XY, but with sex chromosome variations (mosaic and “normal” individuals, and classical and giant X chromosome) that represent an extreme case of intraspecific polymorphism. *Acomys nuguri* could be in the middle of a transition between the ancestral XX/XY system and a system with a single giant X, but this species clearly needs to be examined more thoroughly before any conclusion is drawn.

A few more species are suspected to have unusual sex-determining systems. (i) *Mus triton*, a close relative of the African pygmy mouse, in which convincing G-banded karyotypes of males and females from Burundi were both described as X0 [141]. (ii) The Cabrera vole *Microtus cabrerae*, which has giant asynaptic sex chromosomes [142], and is known for carrying multiple X-linked polymorphic copies of *Sry* [143]. In 1988, four fertile XY females were described in a single population [144]. However, despite extensive search, no new sex-reversed female has since been identified. (iii) Species from two bat genera, *Epomophorus* and *Epomops*, might have an XX/X0 sex-determining system (with X0 males) [145,146]. However, the standard karyotypes (i.e., with no banding) and the low resolution of meiotic analyses do not permit us to discard the possibility that the Y chromosome has fused with an autosome rather than disappeared.

To finish, even if this does not constitute a modification of the sex chromosome system, we would like to mention the curious case of constitutive hermaphrodism found across European moles and other talpid species, and best described in the Iberian mole *Talpa occidentalis.* In these species, while XY individuals develop normal male sexual features, the gonads of XX individuals develop as ovotestes, composed of a small portion of functional ovarian tissue and of a generally large-sized dysgenic testicular tissue [147,148,149,150,151]. The ovarian portion contains follicles and represents the fertile component of the gonad. The testicular portion shows no germ cells, numerous resembling testis cord structures with embryonic Sertoli-like cells but that do not produce SOX9 or AMH, and Leydig cells producing high level of testosterone, responsible for the partial masculinization of females [150,152].

## 5. Discussion

Therian mammals have among the oldest and most conserved sex chromosomes known to this day. However, as illustrated in this paper, there are a few exceptions, and a handful of species harbor unusual sex-determining systems. Though our understanding of these systems is increasing, many questions remain. In particular, one crucial question is still unanswered: how did these species independently manage to escape the evolutionary trap that seems to characterize highly differentiated and specialized sex chromosomes? Finding an answer to this question will certainly further our comprehension of the mechanisms involved in transitions among sex-determining systems, and help explain why certain taxa experience frequent sex chromosome turnovers, while others seem stuck with their old sex chromosomes. A good place to start might be to identify features that are shared among these abnormal mammals.

### 5.1. Curious Similarities

Firstly, it is interesting to note that mammals with unusual sex-determining systems are exclusively rodents (if we exclude the sloths and bats mentioned in the previous section, in which the atypical nature of sex determination remains to be confirmed). There are several non-exclusive hypotheses that may explain this co-occurrence, that were discussed elsewhere [13,41]. Among them, it was proposed that the recurring emergence of novel sex-determining systems might be more common in rodents as their genomes have among the greatest intra- and inter-specific plasticity of all mammals, including an ultra-dynamic karyotypic evolution [153,154]. This suggests that their genomes might be more tolerant or prone to change. Furthermore, this review highlights that these species share intriguing genomic similarities, which we believe might be central puzzle pieces to understand the evolution of these systems.

Mammals seem to have a limited number of options to break loose from their 150-million-year-old sex chromosomes. The species described in this paper fall into two categories: first those in which a feminizing mutation, dominant over *Sry*, arose, leading to an XX, XX*, and X*Y/XY system; and second, those in which the Y chromosome was lost. Species in each group share common features, that were discussed above; here we examine other curious genomic similarities, summarized in Table 1. In particular, a fair share of these species displays sex chromosome features that are extremely rare among other eutherian mammals: (i) asynaptic and achiasmatic behavior of sex chromosomes during male meiosis (six species), (ii) sex-autosome translocations (five species), and (iii) sex chromosome drive (i.e., non-mendelian transmission of sex chromosomes; at least 6 species).

The first two features are thought to be rare due to their negative impact on meiotic processes. (i) Synapsis and formation of chiasmata during meiosis is deemed essential for a proper segregation of chromosomes, and failure to synapse can generate meiotic disruptions and gamete aneuploidy [26]. This has been argued to be the reason why X and Y chromosomes in most eutherian mammals have conserved a small region of homology (the PAR), which allows the two chromosomes to pair, recombine, and form a chiasma during male meiosis [155,156]. Nevertheless, the X and Y chromosomes have lost their ability to synapse and recombine in few mammalian lineages (marsupials [157,158], some gerbils [159], some voles and lemmings [160,161], and African pygmy mice [69]), and in most of them, special meiotic structures have evolved to mediate faithful segregation of sex chromosomes (e.g., dense plate in marsupials; [158]). Among species with unusual sex-determining systems, *M. schisticolor*, *D. torquatus*, *L. mandarinus*, *M. minutoides*, *M. triton*, and *M. cabrerae* belong to lineages in which X and Y chromosomes do not synapse during meiosis [18,142,161,162,163]. Additionally, in *E. fuscocapillus*, the only mole vole with XX/XY sex chromosomes, the synapsis of sex chromosomes is altered: the X and Y chromosomes undergo early desynapsis during late pachytene/early diplotene, a feature which could also lead to incorrect chromosome segregation and formation of zygotes with an X0 complement [96,102].

(ii) Sex-autosome translocations are considered one of the most deleterious chromosomal rearrangements, generating important perturbations in gametogenesis and gene expression, notably due to conflicting replication requirements between sex chromosome and autosomes, the spread of X inactivation into the autosome, and the intrusion of autosomal material into the sex body [164]. Notwithstanding, sex-autosome translocations have been described in a over twenty mammalian genera [165]. Among the species with unusual sex determination systems, *D. torquatus*, *M. minutoides*, and *L. mandarinus*, as well as *C. hoffmanni* and *M. triton* display sex-autosome translocations [42,64,67,84,133,141]. Additionally, a sex-autosome translocation was reported in *Tokudaia muenninki*, the sister species of the two Japanese rats with no Y chromosome [124].

Finally, (iii) an unbalanced, non-mendelian transmissions of sex chromosomes is an extremely rare phenomenon. A skewed transmission of sex chromosome has only been described in a handful of species, mostly insects. As they cause deviations from the evolutionary stable 1:1 male:female ratio, and generate conflicts among genomic compartments with different transmission patterns, sex chromosome drivers tend to be strongly counter-selected. In mammals, the only known case of sex chromosome drive in a species with a standard XX/XY system is found in the house mouse [166,167]. All other cases have been described in rodents with unusual sex-determining systems, i.e., *M. schisticolor*, *D. torquatus*, *A. azarae*, *M. minutoides*, *L. mandarinus*, as well as *M. oregoni* [42,48,58,78,86,129]. 

The co-occurrence in the same group of species of several of these rare sex chromosome features is exceptional, and thus likely not fortuitous. 

### 5.2. What Causes the Evolution of These Weird Systems?

The first two common features mentioned in the previous part: asynaptic/achiasmatic behavior of sex chromosomes and sex-autosome translocations are directly linked to meiotic processes. Interestingly, they are reminiscent of the predictions of the fragile Y hypothesis [168,169]. According to this hypothesis, high rates of Y aneuploidy during male meiosis can select for (i) sex-autosome translocations, to restore an accurate segregation of sex chromosomes, (ii) changes in the segregation mechanism of the X and the Y (i.e., from synaptic to asynapsic/achiasmatic) and finally, (iii) gene movement off the Y chromosome, which can ultimately cause the complete loss of the Y. In their model, the authors mention the reduction in the size of the PAR as the mechanism triggering aneuploidy (conveniently, rodents have among the smallest PARs found in mammals [170], providing an additional hypothesis to explain why unusual sex-determining systems are found solely in rodents). We argue that other factors known for causing meiotic instability, for instance sex chromosome asynapsis, could also select for the loss of the Y (as long as no compensatory mechanism evolves). Thus, we propose that the prevalence of meiotic features associated with defective segregation of sex chromosomes among species with unusual sex-determining systems might not be a coincidence, i.e., that meiotic instability might be responsible for modifications in the mechanism of sex determination. 

Another model has recently been proposed to account for the perseverance of the mammalian XY system: the persistent Y model [87]. Under this model, the presence of certain Y-linked genes severely obstructs Y loss. These genes, so-called “meiotic executioners”, must comply with the two following conditions: they must be essential for successful meiosis, and their transposition to an autosome must be counter-selected. *Zfy* genes, found on all eutherian Y chromosomes [89], meet these two requirements: their expression triggers meiotic sex chromosome inactivation (MSCI); and their insertion onto an autosome causes meiotic arrest, as they need to be subjected to MSCI for meiosis to unfold properly [171,172]. As argued by the authors of the persistent Y model, the only place in the genome *Zfy* genes (and potentially other yet to be characterized meiotic executioners) can be translocated to without causing any damage is the X chromosome, which also undergoes MSCI. As it turns out, in *Ellobius* mole voles, *Tokudaia* spiny rats, and in the creeping vole *M. oregoni*, which have all lost the Y chromosome, *Zfy* was translocated to the X chromosome [95,115,129]. The fragile Y and persistent Y are compelling models to justify the stability of the XY system in therian mammals, but also the loss of the Y in certain lineages. Both place meiosis as a potentially key component to explain the evolution of certain unusual sex-determining systems, namely those in which the Y chromosome was lost.

For now, there is no evidence of a link between meiotic impairments and the evolution and spread of feminizing X* chromosomes. Nevertheless, the evolution of XY females has received a lot of attention from theoreticians, and several factors have been proposed as putative evolutionary drivers. In the late seventies and eighties, work on the two lemmings *M. schisticolor* and *D. torquatus* motivated multiple researchers to investigate the evolution of these peculiar XX, XX*, and X*Y/XY systems using population genetics mathematical models [47,173,174,175,176,177,178]. Briefly, it was proposed that the spread and long-term maintenance of the X* could be due to its effect on sex ratio. Certain characteristic features of these species’ population dynamics, namely local mate competition or interdemic selection, are known to favor individuals that produce more daughters than sons, which makes carrying an X* advantageous, and therefore, would greatly help a feminizing mutation spreading. Alternatively, more recent theoretical analyses have shown that sex chromosome drive (found in one shape or another in all species with XY females; Table 1) could also favor transitions among sex-determining system [179,180,181]. For instance, in X*Y females of *M. schisticolor*, the X* chromosome has a 100% transmission advantage. Assuming that the gene responsible for this drive is X-linked and predates the X*, the newly evolved feminizing mutation would have gained a great selective advantage from being linked to the driving allele (a form of genetic hitchhiking). In these conditions, the X* could have spread even if the X*Y females initially had a poor fitness. Nevertheless, that fact this X*-drive is not found in other species with X*Y females suggests that it more likely evolved secondarily, as a way to reduce the proportion of non-viable YY embryos produced. According to another model [179], feminizing mutations can be selected as a response to Y chromosome drive, as the later induces a shift towards a male-biased sex ratio, and the former turns certain genetic males into females. Such drivers are found in *D. torquatus*, *M. minutoides*, and *L. mandarinus*, and are also suspected in *Akodon* species [48,58,78,86]. The great advantage of this model is that it allows for X* chromosomes to spread even if X*Y females were initially less fit than the two other types of females [78,179], which is likely the case, as XY females in other mammals tend to have a poor fertility if not sterile. Nevertheless, models also show that Y-drive can very well evolve after the establishment of an X*, which makes the causal connection between the two uncertain [78,86].

Despite the fact that several convincing hypotheses have been proposed to account for the evolution of bizarre mammalian sex chromosome systems, no case has been undoubtedly resolved. Research in that area should nevertheless be encouraged, as these mammals make valuable models to test the different hypotheses proposed to explain transitions among sex-determining systems [182].

## 6. Conclusions

Although unusual sex determination systems in mammals have received a slight increase in attention in the last ten years (e.g., [60,95,129]), they remain unexpectedly poorly studied. In particular, the search for the genetic bases of these newly evolved systems has been surprisingly left aside: in all species mentioned, the identity of the gene(s) that have taken *Sry*’s role of master sex determiner remains unknown. Surprisingly, the mammalian sex-determining cascade is still far from being completely understood, as many key player genes are yet to be characterized (review in [183]). To date, a large amount of knowledge (including the identification of *Sry*) has been accumulated via analyses of human patients or laboratory mice with, respectively, pathological or genetically manipulated sex reversal (e.g., [184,185]). The study of species with XY females has already proven that the mammalian X carries one or several genes with still unknown function but essential for male differentiation program, so undoubtedly, some of the next major advances in the field will come from work on these rodents, that are far from being models. These species also offer evolutionary variation that challenges the classical paradigm of sex chromosome evolution. They can therefore be exploited to gain a better understanding of the general principles that normally govern sex chromosome evolution, function, and organization in mammals. For instance, recent cytogenetic studies on several of these species has improved our comprehension of the complex behavior of mammalian sex chromosomes during meiosis, and has provided clues on the still poorly understood early steps of sex chromosome evolution [18,19]. Finally, as they have partially escaped the evolutionary trap that characterizes old sex chromosome systems, they also represent prime candidates to further our understanding of the mechanisms involved in transitions between sex-determining systems.

## Figures and Tables

**Table 1:** Species with confirmed unusual sex-determining systems, and presence/absence of rare genomic features.

Species	Females	Males	Asynaptic Sex Chromosomes in Male Meiosis	Sex-Autosome Translocation	Biased Transmission of Sex Chromosomes
*Myopus schisticolor*	XX, XX*, and X*Y	XY	Yes	No	X*-drive in X*Y females
*Dicrostonyx torquatus*	XX, XX*, and X*Y	XY	Yes	Multiple	Y-drive in males
*Akodon* sp.	XX, XX*, and X*Y	XY	No	No	Suspected Y-drive in males and X*Y females
*Mus minutoides*	XX, XX*, and X*Y	XY	Yes	Multiple	Conditional male drive
*Lasiopodomis mandarinus*	XX, XX*, and X*Y	XY	Yes	Multiple	Conditional male drive
*Ellobius lutescens*	X0	X0	NA	No	Unknown
*Ellobius tancrei/talpinus/alaicus*	XX	XX	No	No	Unknown
*Tokudaia osimensis/tokunoshimensis*	X0	X0	NA	No, but yes in sister species *T. muenninki*	Unknown
*Microtus oregoni*	X^M^0	X^P^X^M^	NA	No, but possible X–Y fusion	X^M^-drive in females X^M^ loss in males
Other species			*Microtus cabrerae*, *Mus triton*	*Choloepus hoffmanni, Mus triton*	Unknown

## Data Availability

Not applicable.
